# False Alarm: When Dropped Gallstones Mimic Malignant Recurrence. A Case Report and Literature Review

**DOI:** 10.1155/crog/5565976

**Published:** 2026-01-18

**Authors:** Nedaa Obeidi, Michael Egan, Michael E. Kelly, Feras Abu Saadeh

**Affiliations:** ^1^ Department of Obstetrics and Gynaecology, Trinity College Dublin, Dublin, Ireland, tcd.ie; ^2^ Department of Surgery and Anesthesia, Hermitage Medical Clinin, Dublin, Ireland; ^3^ Department of Surgery, Trinity St. James′s Cancer Institute, Dublin, Ireland; ^4^ Department of Gynecology, St. James′s Hospital, Dublin, Ireland

## Abstract

Dropped gallstones are a known complication of laparoscopic cholecystectomy, occurring in up to 40% of cases. While often considered benign, they can lead to complications like abscess formation and peritoneal adhesions. In cancer patients, dropped gallstones may mimic disease recurrence, leading to unnecessary diagnostic procedures and patient distress. We report the case of a 63‐year‐old woman with a history of leiomyosarcoma, previously treated with surgical resection. She subsequently underwent laparoscopic cholecystectomy for acute cholecystitis. Ten months later, she presented with nonspecific upper abdominal symptoms, and imaging indicated peritoneal nodularity suspicious for malignancy. A CT‐guided biopsy was inconclusive, prompting diagnostic laparoscopy revealing multiple dropped gallstones with granulomatous inflammation but no evidence of malignancy. The patient remained symptoms free after retrieval, with no further radiological abnormalities on follow‐up imaging. This case highlights the diagnostic challenges posed by dropped gallstones, particularly in oncology patients where they can be mistaken for peritoneal metastases. Surgeons should be meticulous to retrieve all gallstones during laparoscopic cholecystectomy, and radiologists should include dropped gallstones in the differential diagnosis of new intra‐abdominal lesions in postsurgical patients. Awareness of this phenomenon can prevent unnecessary interventions and patient anxiety.

**Summary**

• Dropped gallstones are a common complication of laparoscopic cholecystectomy.
• Dropped gallstone could cause serious complication and every effort should be made to retrieve it at the time of surgery.
• In a setting of cancer diagnosis, dropped gallstone may mimic cancer metastasis.

## 1. Introduction

Dropped gallstones in the peritoneal cavity are a frequent complication of laparoscopic cholecystectomy, can happen in up to 40% of cases [1]. It is more likely to happen in complicated cases with an inflamed gallbladder with multiple stones [2]. Typically, this results from gallbladder perforation during dissection and extraction. Generally, dropped gallstones should be meticulously removed; however, this may not occur or be identified, and documentation can be inadequate. Dropped gallstones are thought to be insignificant and do not carry a risk to the patient and it was not mandatory to retrieve them [3]. However, they can result in many complications, the most common one being the intraperitoneal abscess [4]. In the setting of cancer patients, these dropped gallstones may mimic a recurrence of the disease. That creates huge anxiety for the patients and makes their management very challenging [5].

## 2. Case Presentation

We present a case of a 63‐year‐old woman, a mother of three children, all vaginal delivery. She had menopause around the age of 52 and never used hormone replacement therapy. She was an ex‐smoker and had moderate alcohol consumption. Her medical history is limited to hypothyroidism and asthma for which she was treated with inhalers and Eltroxin.

The patient presented initially 5 years ago with postmenopausal bleeding to a general gynecologist and had an endometrial biopsy which was benign; however, a pelvic MRI demonstrated an enlarged uterus with multiple fibroids. The largest was 9 × 7.3 × 6 cm submucosal. The patient was offered a hysterectomy; she declined the medical advice and insisted on myomectomy. An open myomectomy was performed, and she was diagnosed with leiomyosarcoma. The histology confirmed hypercellular spindled cell tumor with necrosis, atypia, and mitosis of 29/10 HPF. Immunohistochemistry shows strong positivity for SMA, ER and PR receptors, desmin, and AE1AE3. The finding was consistent with leiomyosarcoma extending to the resection margin.

The patient′s case was referred to our oncology center and was discussed at the gynecology cancer multidisciplinary meeting. A staging CT scan did not show any evidence of distal metastasis. The recommendation from the meeting was for completion surgery in view of the size of the tumor and the positive disease at resection margin. The patient had debulking surgery. The uterus was found to be densely adherent to bowel at two sites and she had total hysterectomy bilateral salpingo‐oophorectomy with resection of terminal ileum and rectosigmoid with primary ileo‐ileal anastomosis and colorectal anastomosis. There was no residual tumor in the final specimen. The final stage was IB and she did not have any adjuvant treatment.

**Figure 1:**
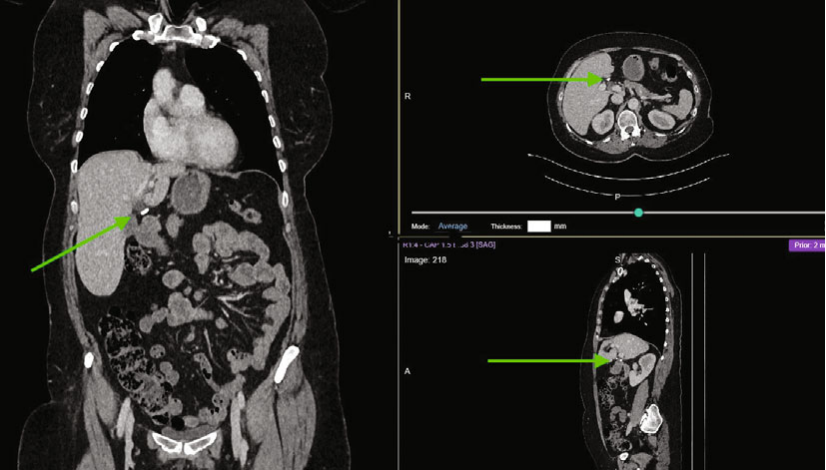
Preoperative CT TAP scanning showing radio opaque lesion in the upper abdomen suspicious for cancer recurrence.

Patient was followed up at our oncology clinic with clinical exam and chest X ray 3 months initially and then every 6 months. Patient recovered very well from her surgery, and she did not show any sign of disease recurrence. Eighteen months post primary surgery, the patient was admitted to hospital with an inflamed gallbladder. The ultrasound described multiple gallbladder stones ranging in size from 2 to 22 mm, with wall thickening. The patient underwent a laparoscopic cholecystectomy. Due to significant inflammation, the procedure was complex, with gallbladder rupture and spillage of stones. A washout was performed, and an attempt was made to retrieve the spilled stones. The patient recovered well following the procedure. Ten months post cholecystectomy, she presented with nonspecific upper abdomen symptoms for which she had a CT scan. The findings on the scan were suspicious of cancer recurrence with multiple ill‐defined foci of peritoneal nodularity around the liver ranging in size from 0.7–2.3 cm (Figure 1). The scan was discussed at the gynecological cancer MDM, and the recommendation was for CT guided biopsy.

**Figure 2:**
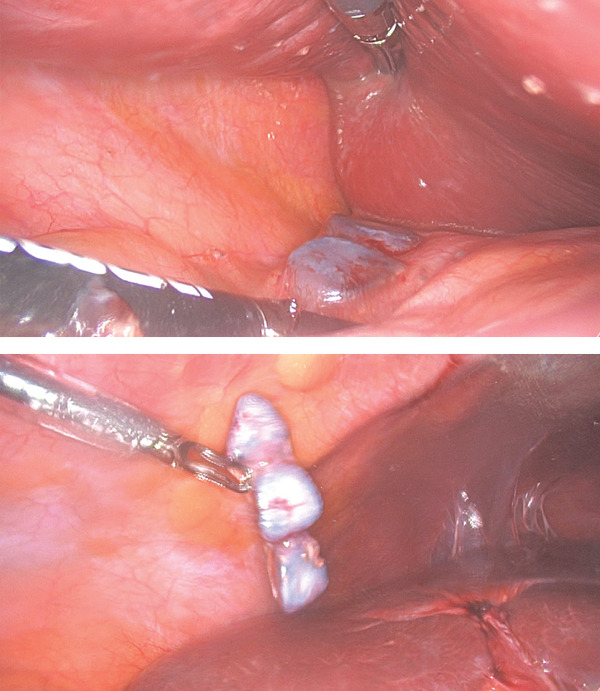
A laparoscopic finding of dropped gallstones.

**Figure 3:**
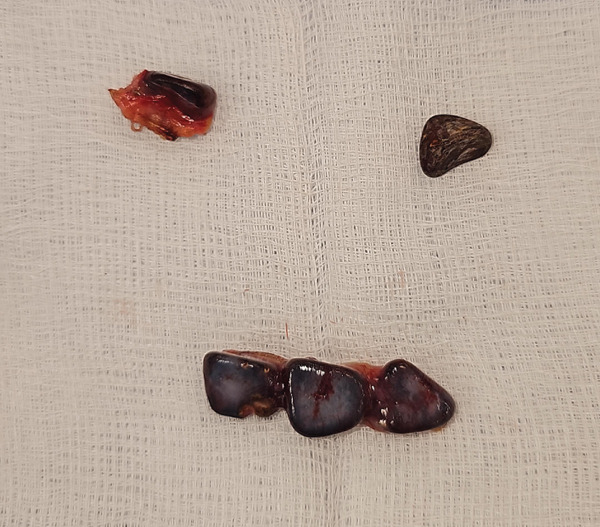
Macroscopic view of the retrieved gallstone.

An ultrasound‐guided biopsy was attempted but was not possible, as the previously noted soft tissue on CT was not visible on ultrasound. Therefore, we proceeded to a diagnostic laparoscopy. During laparoscopy, dropped gallstones were found in front and behind the liver (Figure 2), and no sign of malignancy was found. Histology showed fibrofatty tissue with multiple foci of granulomatous inflammation surrounding yellow pigmented material (probably bile). Five yellow/brown gallstones were retrieved (Figure 3). The patient had a CT 6 months post the laparoscopy, and no abnormalities were noted.

## 3. Discussion

Dropped gallstones after laparoscopic cholecystectomy are common. It can happen in up to 40% of cases [2]. In most cases, it is a problem that tends to be ignored at the time of primary surgery as it is thought to be harmless. However, in 4%–8% cases, it may result in serious harm like abscess, migration, and adhesion [6, 7]. Dropped gallstones are recognized as a more frequent issue in laparoscopic cholecystectomy compared with open surgery due to the unique technical and physical constraints of the minimally invasive approach, including limited tactile feedback, instrument‐induced perforations, and extraction through small ports [8–10]. An inflamed gallbladder is edematous and friable, making it more prone to tearing during laparoscopic traction, which increases gallstone spillage. Inflammation also distorts anatomy and makes dissection more difficult, further predisposing to perforation and stone loss [8]. The leakage of bile at the time of cholecystectomy is thought to increase the risk of complications. Zorlouglu in an animal experiment has shown that an implanted gallbladder stone in the rat peritoneal cavity without bile did not cause any abscess or adhesion. When they add the bile, that increases the risk of intraperitoneal complications [11]. In our patient, the histopathological examination of the retrieved stone did show evidence of bile; clinically, the patient had an empyema at the time of her surgery. The two factors of bile leakage and infection may play a significant role in causing the adhesion and the upper abdomen symptoms.

Most of the cases are presented in the first year post surgery; however, some cases are reported more than 15 years after the index surgery [12]. It can present in different ways: abdominal pain, pelvic pain, fistula, or intestinal obstruction. In a meta‐analysis of 71 cases, 9 patients (12.7%) presented with an abdominal mass resembling metastasis [13]. Dropped gallstones can form inflammatory nodules that radiologically resemble peritoneal metastases. Any new lesion on a follow‐up scan in a cancer patient raises suspicion of recurrence, creating significant diagnostic uncertainty in oncology care. This results in an extensive investigation and significant physical and psychological trauma to patients, and in some cases, very major unnecessary abdominal surgery [14].

There are no unique diagnostic modalities to diagnose dropped gallstone with confidence. The radiological finding depends on the stone biochemical component. Stone that is made up of calcium will appear as a high attenuation calcified lesion. Stone that is high in cholesterol and low in calcium will appear as low attenuating lesion. However, a significant proportion of stones are isoattenuating relative to surrounding bile and may be radiologically occult on CT [15]. The stone in our patient was clearly visible on CT but was not visible on US, which creates an issue in the diagnostic work‐up of the patient.

## 4. Conclusion

Dropped gallstones were an uncommon incident after open cholecystectomy, but have significantly increased with the popularity of laparoscopic cholecystectomy, especially when dealing with large and inflamed gallbladders. It is usually harmless, but it has the potential to cause serious harm, and every effort should be made to retrieve them at the time of primary surgery. In cases of cancer, it can be difficult to rule out the possibility of malignancy without surgically removing them.

## Consent

Written informed consent was obtained from the patient for publication of this case report and accompanying images. A copy of the written consent is available for review by the editor‐in‐chief of this journal on request.

## Conflicts of Interest

The authors declare no conflicts of interest.

## Author Contributions

Dr. Nedaa Obeidi: investigation, writing—original draft, writing—review and editing, visualization. Dr. Michael Egan: resources, writing—review and editing. Prof. Feras Abu Saadeh: conceptualization, resources, supervision, project administration, writing—review and editing.

## Funding

No funding was received for this manuscript.

## Data Availability

The data that support the findings of this study are available on request from the corresponding author. The data are not publicly available due to privacy or ethical restrictions.
